# Efficacy of sodium new houttuyfonate against *Aspergillus flavus*: insights from *in vitro* and *in vivo* models of invasive pulmonary aspergillosis

**DOI:** 10.3389/fphar.2025.1577561

**Published:** 2025-05-01

**Authors:** Yilin Bao, Dingxian Feng, Jinping Zhang, Xiaoyan Hu, Xinyou Yang, Yingyu Mao, Zhangyong Song

**Affiliations:** ^1^ Public Center of Experimental Technology, School of Basic Medical Sciences, Southwest Medical University, Luzhou, China; ^2^ Department of Otolaryngology - Head and Neck Surgery, The Affiliated Hospital of Southwest Medical University, Luzhou, China; ^3^ Hemodynamics and Medical Engineering Combination Key Laboratory of Luzhou, Southwest Medical University, Luzhou, China

**Keywords:** *Aspergillus flavus*, sodium new houttuyfonate, invasive pulmonary aspergillosis, antifungal agent, *in vivo* activity, inflammatory response

## Abstract

*Aspergillus flavus* is an opportunistic pathogenic fungus that causes invasive aspergillosis in humans. Due to the limited variety and quantity of clinical antifungal drugs and their adverse effects, the development of new broad-spectrum antifungal drugs is urgently required. Our preliminary research showed that sodium new houttuyfonate (SNH) was efficacious against *A. fumigatus* infection, but its effects against *A. flavus* remain unknown. In this study, we used the microdilution broth susceptibility method to determine the antifungal activities of four antifungal drugs and SNH against 12 clinical *A. flavus* strains, *in vitro*. To confirm the therapeutic effect of SNH on *A. flavus* infection, we established a mouse model of invasive pulmonary aspergillosis (IPA) with the nasal drip method. All the strains tested were resistant to fluconazole but sensitive to itraconazole, voriconazole, and amphotericin B. The minimum inhibitory concentration to inhibit the growth of 90% of cells (MIC90) of SNH against the test strains was 64–128 μg/mL. After the IPA mouse model was treated with SNH, the expression of genes encoding interleukin 6 (IL-6), IL-1β, and tumor necrosis factor α was significantly reduced. SNH also reduced the fungal load in the mouse lung, the extent of pathological damage, and the neutrophil/lymphocyte ratio in the blood. These findings indicated the potential utility of SNH in the treatment of *A. flavus* infections.

## 1 Introduction

Invasive pulmonary aspergillosis (IPA), the commonest pulmonary fungal disease, is an opportunistic fungal infection that occurs in immunocompromised patients (Blanchard et al., 2018). With the prevalence of immunocompromised populations, the incidence of patients with IPA has gradually increased and with a mortality rate of 18.5% (Denning, 2024). Recent studies have shown that about one-third of the 3.23 million deaths annually worldwide are generally related to *Aspergillus* infections (Denning, 2024; Liu et al., 2024). *Aspergillus flavus*, an opportunistic pathogenic fungus and the primary cause of IPA, causes about 10% of IPA infections (Rudramurthy et al., 2019), and generates the secondary metabolite aflatoxin, which is strongly hepatotoxic and teratogenic (Calvo et al., 2002; Amaike and Keller, 2011; Sebők et al., 2016). In addition, it had demonstrated that *A. flavus* has adept in infection ability. Moreover, compare with that in the healthy mice, it give rise to stronger immunological response in the immunocompetent mice (Anand et al., 2013; Shankar et al., 2024). There are currently three available classes of agents that can be used in clinical practice. Some drugs have obvious adverse effects or limited administration methods. Furthermore, the increasingly serious problem of fungal drug resistance poses a serious challenge to the clinical treatment of pathogenic fungi. Therefore, the development of new antifungal drugs are urgently required (Zhang et al., 2021). Here, we confirm an alternative strategy by studying the antifungal effect of an existing drug.

**FIGURE 1:**
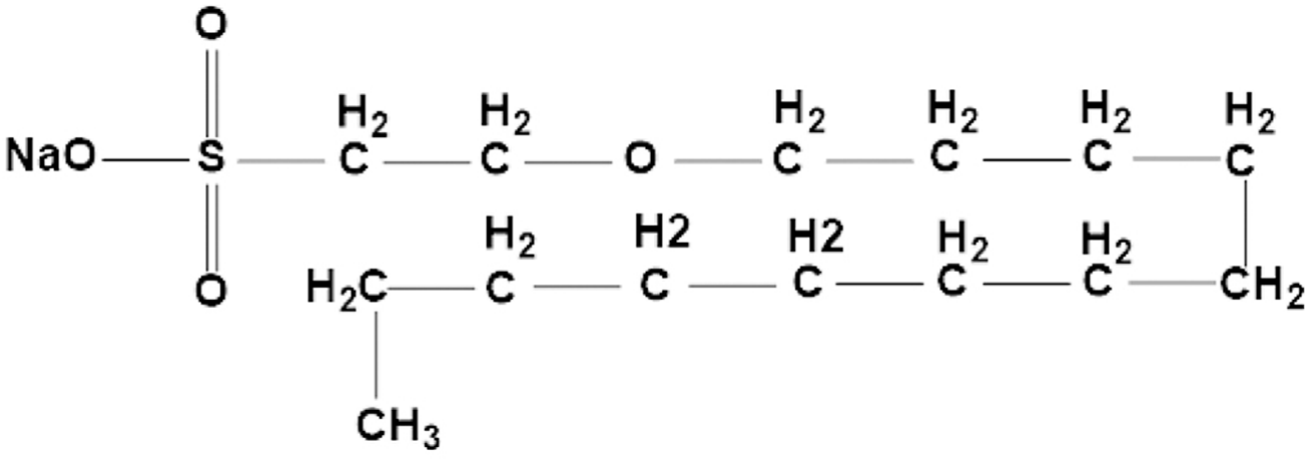
Chemical structure of SNH (sodium new houttuyfonate).

Sodium new houttuyfonate (SNH) (Figure 1), a chemical compound from *Houttuynia cordata*, has various biological and pharmacological activities, including anti-inflammatory (Jiang et al., 2019), antibacterial (Yang et al., 2016), anti*-Candida albicans* (Wu et al., 2020), and anticancer properties (Dai et al., 2021). SNH also promotes the excessive accumulation of reactive oxygen species (ROS), induces mitochondrial damage, and targets the PDK1-AKT-GSK3β pathway, and plays a significant role in the apoptosis of breast cancer cells (He et al., 2023). In clinical, SNH acts as an anti-inflammatory medicine, however, because of its parenteral solutions causing severe allergic reaction in clinical use, the use and approval of injectables are limited (Liu et al., 2021). Nevertheless, our strategy is studying the antifungal effects of SNH with oral dosage forms. Similar to itraconazole, our preliminary research confirmed *in vitro* that SNH inhibited the growth of *A. fumigatus* by interfering with the steroid synthesis pathway and inhibiting ergosterol synthesis in the fungal cell. In addition, its efficacy against *A. fumigatus* infection *in vivo* was demonstrated. Moreover, the SNH also had the antifungal effects in the *A. fumigatus* resistant strains (Zhang et al., 2022). However, no anti-*A. flavus* effect of SNH has yet to be reported.

To further confirm the antifungal spectrum of SNH, in this study, we investigated the antifungal activity of SNH against 12 clinical *A. flavus* strains *in vitro*. Based on fungal burden assays, routine blood tests, histopathological analyses, and quantitative real-time (qPCR) analysis, we demonstrated that SNH was efficacious against *A. flavus* strains in a mouse model of IPA. Our results indicated that SNH was a promising agent for the treatment of *A. flavus* infections.

## 2 Materials and methods

### 2.1 Strains and drug preparation

Twelve clinical *A. flavus* isolates were collected from of the sputum or bronchoalveolar lavage fluid of clinical patients (Table 1) and stored in the Laboratory for Pathogenic Fungal Infection Prevention Research of Southwest Medical University, Luzhou, Sichuan, China. The standard strain *A. fumigatus* AF293 was used as the control strain and was stored at −80°C. Before each experiment, the test stains were grown on potato dextrose agar (PDA) for 5 days at 37°C. All drugs, including fluconazole (FLC), itraconazole (ITR), voriconazole (VRC), and amphotericin B (AmB), were purchased from Macklin Biochemical Co., Ltd. (Shanghai, China). SNH was obtained from Fengyao Tonghui Chemicals Co., Ltd. (Wuhan, China). In accordance with previous studies (Zhang et al., 2022; Zeng et al., 2023), AmB, FLC, ITR, and VRC were dissolved in dimethyl sulfoxide to prepare stock solutions with concentrations of 5.12 mg/mL, and SNH was dissolved in sterile distilled water containing 0.05% Tween 80 to prepare the stock solution at a concentration of 3.00 mg/mL. All the test drug stock solutions were stored at −20°C.

**TABLE 1:** The minimum inhibitory concentration of 13 test strains to antifungal agents.

Isolate strains	FLC (μg/mL)	VRC (μg/mL)	ITR (μg/mL)	AmB (μg/mL)	SNH (μg/mL)
AF293	256	<0.5	<0.5	<0.5	128
S39	>256	<0.5	<0.5	2	128
S49	>256	<0.5	<0.5	1	128
S51	256	<0.5	<0.5	<0.5	128
S51	>256	<0.5	<0.5	2	64
S52	>256	<0.5	<0.5	2	128
S54	>256	<0.5	<0.5	1	128
S60	>256	<0.5	<0.5	1	128
S64	>256	<0.5	<0.5	4	64
S72	>256	<0.5	<0.5	1	128
S73	256	<0.5	<0.5	<0.5	128
S122	>256	<0.5	<0.5	2	128
S124	>256	<0.5	<0.5	4	128

Note: FLC, fluconazole; ITR, itraconazole; VRC, voriconazole; AmB, amphotericin B; SNH, sodium new houttuyfonate.

### 2.2 Susceptibility to antifungal agents

The susceptibility of *A. flavus* to antifungal agents was determined with the microdilution broth susceptibility assay, according to Clinical and Laboratory Standards Institute (CLSI) document M38-A2 (2008) (Espinel-Ingroff et al., 2010). Briefly, conidia were resuspended in RPMI-1640 medium at a final concentration of 1.0 × 10^5^ cells/mL and incubated in 96-well microtiter plates at 37°C for 48 h. The concentrations of the antifungal agents ranged from 0.25 to 32 μg/mL for VRC, ITR, and AmB, from 4 to 256 μg/mL for FLC, and from 2 to 1,024 μg/mL for SNH. The minimum concentration required to inhibit the growth of 90% of cells (MIC_90_) was determined according to CLSI document M38-A2 (Espinel-Ingroff et al., 2010). All experiments were repeated six times.

### 2.3 Antifungal activity of SNH *in vivo*

**FIGURE 2:**
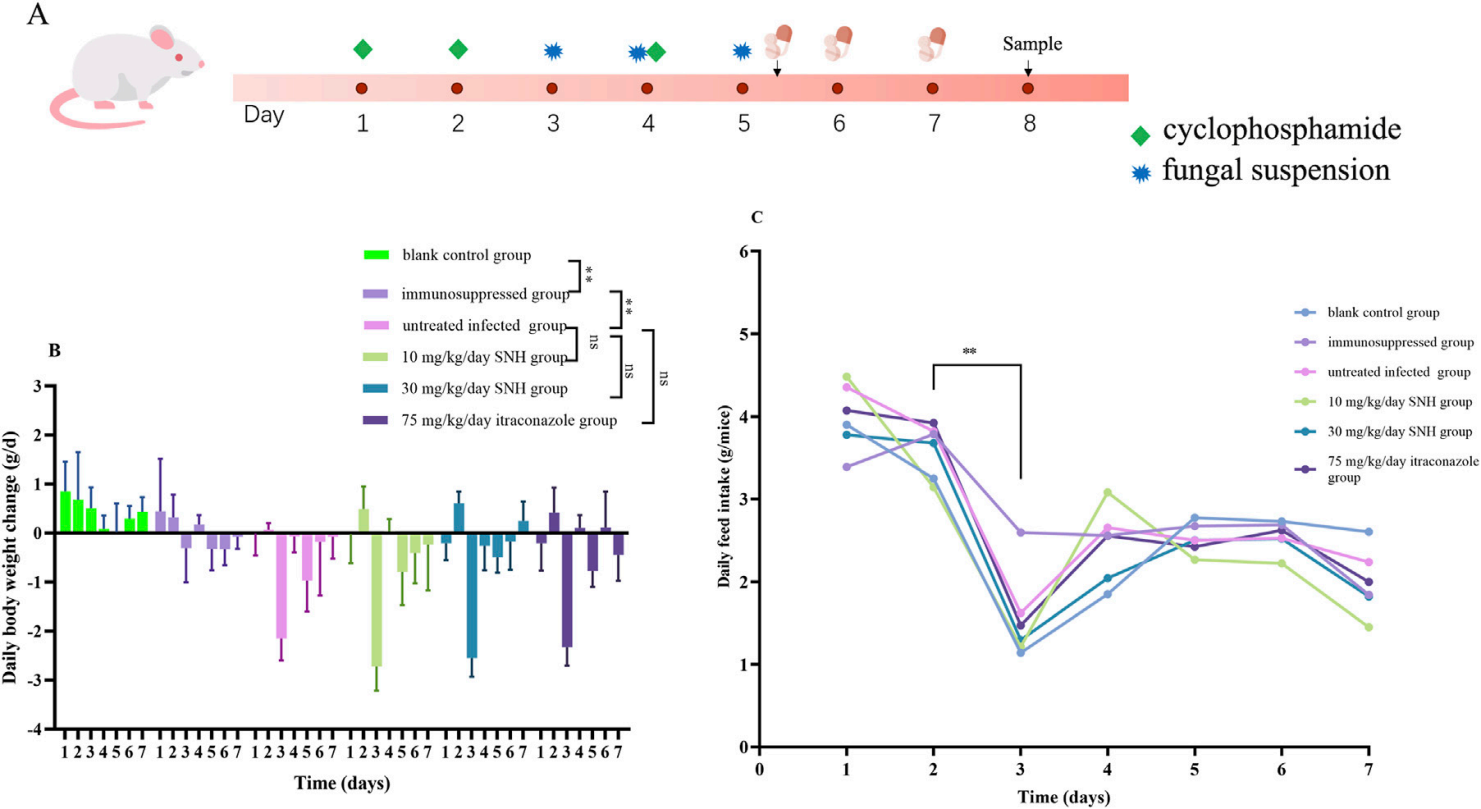
Mouse model and changes in bodyweight and feed intake. **(A)** Mouse model of invasive pulmonary aspergillosis. Six mice were sacrificed in each group for experiments. Daily bodyweight changes **(B)** and feed intake **(C)** in the six groups of mice. The six groups were including blank control group, immunosuppressed group, untreated infected group, 30 mg/kg/day SNH group, 10 mg/kg/day SNH group, and 75 mg/kg/day itraconazole group. The blank control group and immunosuppressed group mice immunosuppressed without or with a subcutaneous injection of cyclophosphamide (200 mg/kg/day) for 3 days. The other groups contained immunosuppressed mice were infected by a nasal drip of 30 μL of sterile saline containing 5 × 10^8^ cells/mL *Aspergillus flavus* per mouse and treated for 3 days without or with drugs administered by gastric gavage. Error bars represent standard error of mean of six mice. Data were analyzed with one-way analysis of variance (ns: no significant difference, *p* > 0.05; ^*^ *p* < 0.05; ^**^ *p* < 0.01).

The mouse experimental protocol was approved by the Southwest Medical University Institutional Animal Care and Use Committee (20221102-009). And the Isolate S39 strain was randomly selected to use to infect mice. In accordance with our preliminary research method (Zhang et al., 2022), Male 6–8-week-old C57BL/6J mice (n = 36; Laboratory Animal Center, Southwest Medical University) weighing 20–25 g, were assigned to one of six groups. Blank control group was the untreated blank control group. Immunosuppressed group was mice immunosuppressed with a subcutaneous injection of cyclophosphamide (200 mg/kg/day) for 3 days. Untreated infected group containing immunosuppressed mice was infected by a nasal drip of 30 μL of sterile saline containing 5 × 10^8^ cells/mL *A. flavus* with no drug treatment. 30 mg/kg/day SNH group contained immunosuppressed mice was infected by a nasal drip of 30 μL of sterile saline containing 5 × 10^8^ cells/mL *A. flavus* and treated for 3 days with SNH (30 mg/kg/day) administered by gastric gavage. 10 mg/kg/day SNH group contained immunosuppressed mice were infected by a nasal drip of 30 μL of sterile saline containing 5 × 10^8^ cells/mL *A. flavus* and treated for 3 days with SNH (10 mg/kg/day) administered by gastric gavage. 75 mg/kg/day itraconazole group contained immunosuppressed mice were infected by a nasal drip of 30 μL of sterile saline containing 5 × 10^8^ cells/mL *A. flavus* and treated for 3 days with itraconazole (75 mg/kg/day) administered by gastric gavage. The nasal drip lasted for three consecutive days (Figure 2A). After the successful establishment of the IPA mouse model, the drug treatments were commenced 1 h after infection and were continued for 3 days (Denning et al., 1995; Feng et al., 2021). The basic vital signs of the mice, including daily bodyweight changes and feed intake, were then observed and recorded daily.

Afterward, the mice were humanely euthanized by cervical dislocation after anesthesia. And the fungal burden and a histopathological analysis were used to confirm the efficacy of SNH against *A. flavus* infection. Kidney, liver, lung, and blood samples were collected from all mice after treatment for 3 days. One-half of each collected kidney, liver, and lung sample was randomly homogenized and plated onto PDA. The number of colony-forming units (CFU) per Gram of collected tissue was counted after incubation for 48 h at 37°C. The sectioned lung tissues were fixed in 4% methanol, embedded in paraffin, cut into thin slices, and stained with hematoxylin and eosin (H&E) for microscopic observation. To determine whether the numbers of white blood cells, lymphocytes, or neutrophils differed among the mouse groups, blood samples were collected from the mouse eyeballs and stored in anticoagulant tubes containing ethylenediaminetetraacetic acid (EDTA) before the complete blood cells were counted (Lu et al., 2022).

### 2.4 Reverse transcription (RT)-qPCR assay of transcriptional activity of inflammatory factor genes

To quantify the effects of SNH treatment on the transcriptional activity of inflammatory factor genes, 0.1 g the collected kidney, liver, and lung tissues were homogenized with 800 mL of RNAiso Plus (Takara, Dalian, China). Total RNA was extracted with RNAiso reagent (Takara) and reversed transcribed to cDNA with reagents provided by Takara. The qPCR was performed with Perfect^®^Start Green qPCR SuperMix (TransGene Co., Ltd., Beijing, China). The genes encoding interleukin 6 (*IL-6*), interleukin 1β (*IL-1β*), and tumor necrosis factor α (*TNF-*α) were detected according to the manufacturer’s protocol. Transcripts of the glyceraldehyde 3-phosphate dehydrogenase gene (*Gapdh*) were used as an internal standard. The 2^−ΔΔCT^ method was used to evaluate the relative changes in gene expression (Vandesompele et al., 2002). The primer sequences used are shown in Supplementary Table S1.

### 2.5 Statistical analysis

Each experiment was performed independently six times. Statistical analyses were conducted and graphs drawn with GraphPad Prism 9.0 (GraphPad Software Inc., La Jolla, CA, United States) and outline any post-hoc tests (Student’s t-test) after one-way analysis of variance. Statistical significance was defined as *p* < 0.05.

## 3 Results

### 3.1 Activities of antifungal drugs and SNH against test strains *in vitro*

The MIC_90_ values of the four antifungal drugs (FLC, ITR, VRC, and AmB) and SNH against the 12 test strains are shown in Table 1. Based on CLSI document M38-A2, the results showed that all test strains were resistant to FLC, as determined with the broth microdilution method, whereas all test strains were sensitive to ITR, VRC, and AmB. The MIC_90_ of SNH against the test strains was 128 μg/mL. We randomly selected a clinical *A. flavus* strain (S39) for the subsequent investigations.

### 3.2 General physical signs in mouse model treated with SNH

Compared with the immunosuppressed group, the mice in the IPA group showed weight loss, delayed responsiveness, reduced activity, hair loss, and sneezing (data not shown). After the infected mice were treated with SNH or ITR, the symptoms of reduced activity and delayed responsiveness were relieved and the symptoms of hair loss and sneezing disappeared. Records of the daily weight changes in the mice showed that after the subcutaneous injection of immunosuppressant, the weight gain of the mice slowed and gradually ceased. On day 3, the M group of mice showed a significant reduction in bodyweight after the nasal instillation of the fungal suspension. However, there is no significant relieving of weight loss after treatment with low concentration SNH, high concentration SNH, or ITR by gavage (Figure 2B). The recorded daily feed intakes also showed that the food intake of the IPA group mice decreased significantly on day 3 compared with those of the untreated blank group and the immunosuppressed group. However, compared with the M group mice, there were no significant differences among the low-concentration SNH group, the high-concentration SNH group, and the ITR group (Figure 2C).

### 3.3 SNH reduces the fungal burden and induces blood changes in the mouse model

**FIGURE 3:**
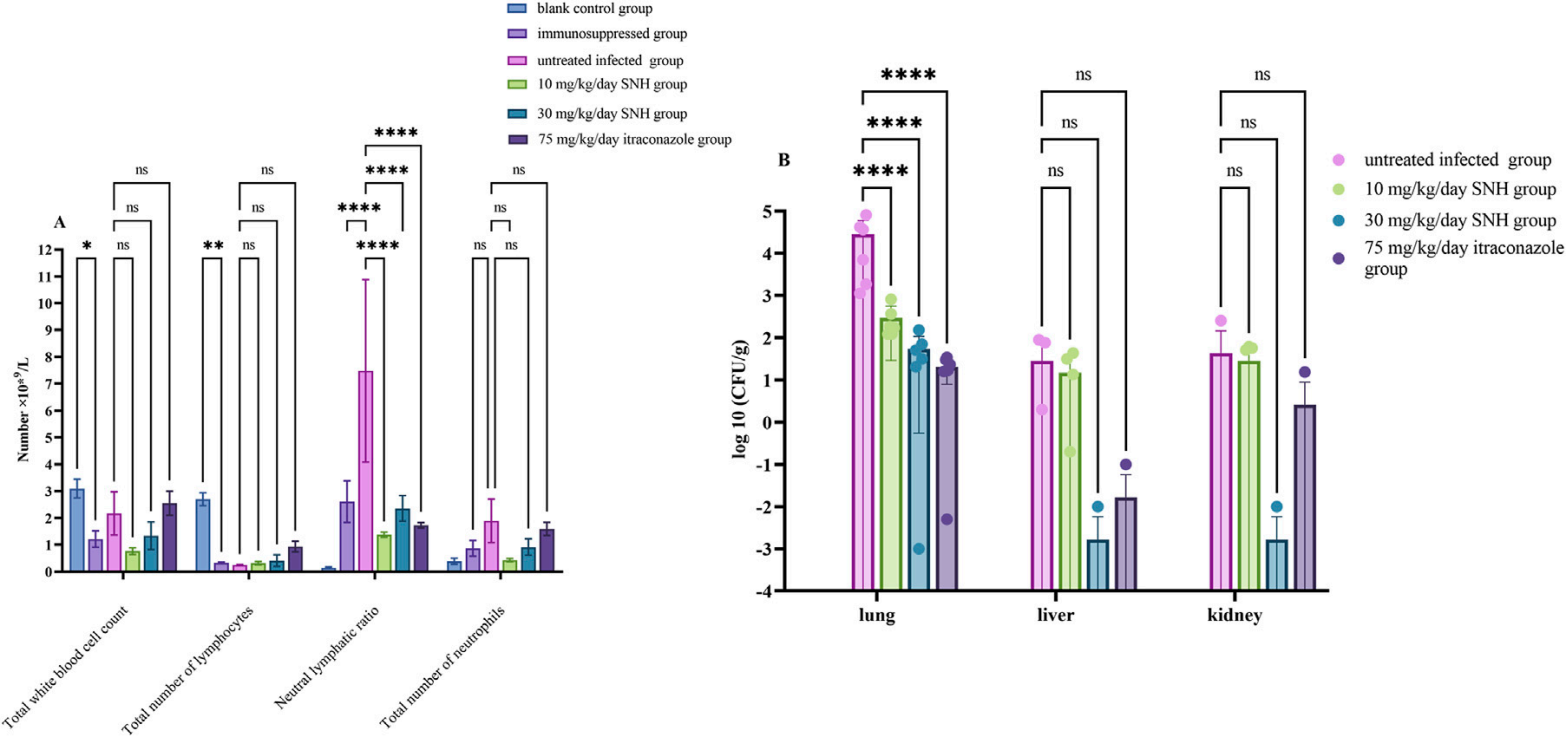
Changes in fungal burden and routine blood parameters in mouse model. **(A)** Results of routine blood cell counts in mice. **(B)** Colony-forming units (CFU) per Gram of collected tissue. Six mice were sacrificed in each group for experiments. Error bars represent standard error of mean of six mice. Data were analyzed with one-way analysis of variance (ns: no significant difference, *p* > 0.05; ^*^ *p* < 0.05; ^**^ *p* < 0.01; ^***^ *p* < 0.001).

The routine blood test showed that the numbers of white blood cells and lymphocytes were lower in the immunosuppressed group than in the blank control group, indicating the successful establishment of the immunosuppressed model mice (Figure 3A). The neutrophil-to-lymphocyte ratio was significantly higher in the infected groups than in the blank control group, suggesting that the IPA infection model was successfully established. Compared with the IPA infection group (untreated infected mice), there were significant reductions in neutrophil-to-lymphocyte ratio in the mice treated with low dose SNH, high dose SNH, or ITR (Figure 3A).

CFU counting showed significantly lower fungal loads in the lungs of the mice treated with low concentration of SNH, high concentration SNH, or ITR than in the untreated infected mice, indicating that both the SNH and ITR treatments reduced the fungal load in the lungs of mice with pulmonary aspergillosis (Figure 3B). However, there was no significant difference in the fungal loads in the liver or kidney tissues among the groups (Figure 3B).

### 3.4 SNH treatment reduces lung tissue lesions in mice

**FIGURE 4:**
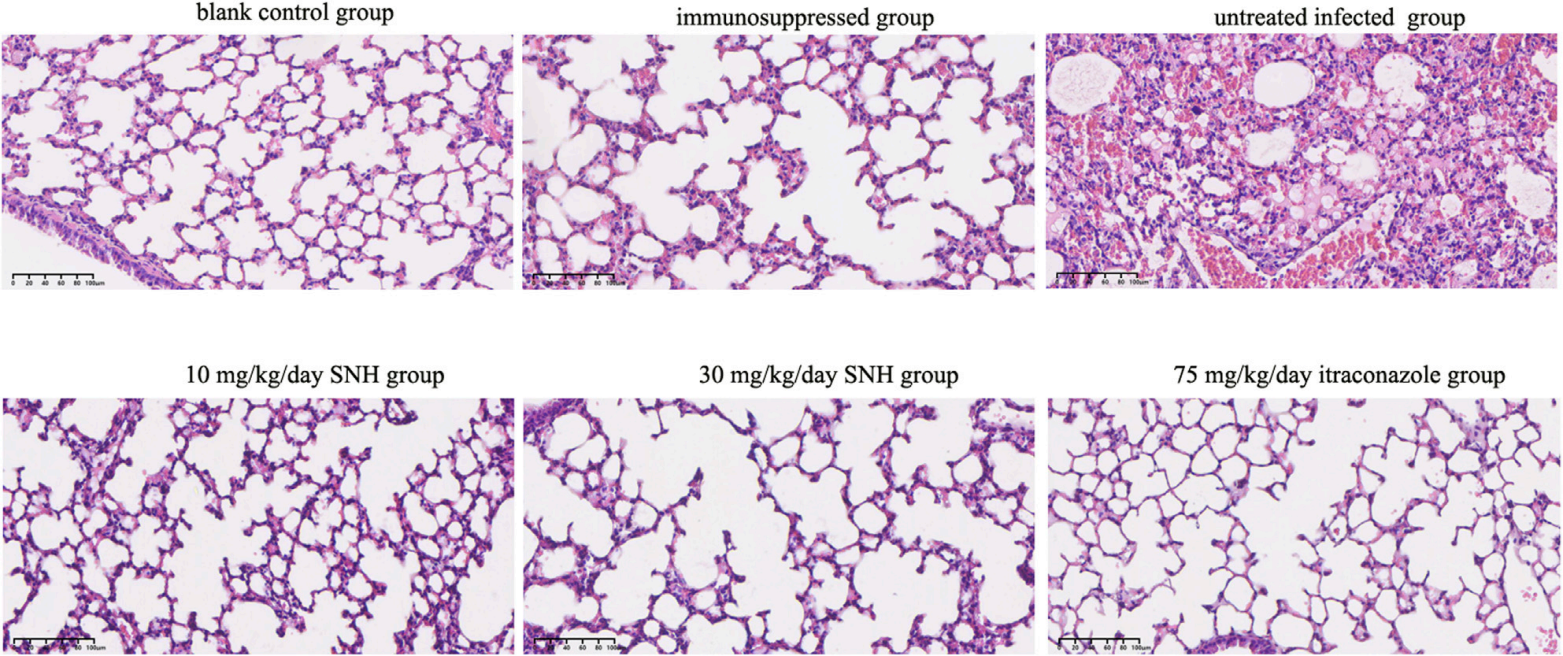
Hematoxylin and eosin (H&E) staining of mouse lung tissues. The six groups were including blank control group, immunosuppressed group, untreated infected group, 30 mg/kg/day SNH group, 10 mg/kg/day SNH group, and 75 mg/kg/day itraconazole group. Bar: 200 μm.

H&E staining of lung tissue sections showed that, compared with the blank and immunosuppressed control groups, the untreated infected mice displayed severe tissue necrosis, the destruction of the normal alveolar structure, increased interstitial width, the infiltration of many inflammatory cells, and bleeding in the lungs (Figure 4). However, the number of pathological areas and the severity of the lesions in the infected mice were significantly reduced after treatment with low concentration SNH, high concentration SNH, or ITR compared with those in the untreated infected mice (Figure 4).

### 3.5 SNH changes the expression of inflammatory factor genes

**FIGURE 5:**
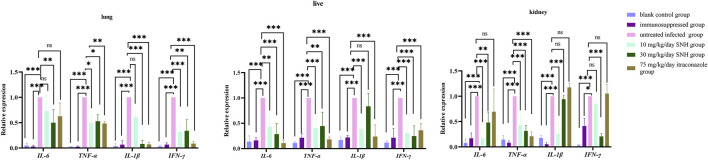
Expression of inflammatory factor genes in collected tissues. Error bars represent standard error of mean of six mice. Data were analyzed with one-way analysis of variance (ns, *p* > 0.05; ^*^ *p* < 0.05; ^**^ *p* < 0.01; ^***^ *p* < 0.001).

Compared with that in the untreated infected mice, the expression of inflammatory factor genes were significantly downregulated in blank and immunosuppressed control groups (Figure 5). In addition, the expression of *IL-1β* was significantly reduced in the lungs of mice treated with high concentration SNH or ITR compared with that of the untreated infected group (Figure 5). It was significantly reduced in the kidneys and livers of mice treated with low concentration SNH and was also significantly reduced in the livers of mice treated with ITR. The expression of *TNF-*α was significantly lower in the lungs, kidneys, and livers of mice treated with low concentration SNH, high concentration SNH, or ITR than in the untreated infected group (Figure 5). The expression of *IL-6* was significantly lower in the livers of mice treated with low concentration SNH, high concentration SNH, or ITR than in those of the untreated infected mice (Figure 5). The expression of *IL-6* was significantly reduced in the lungs of mice treated with high concentration SNH and significantly reduced in the kidneys of mice treated with low concentration or high concentration SNH.

## 4 Discussion

At present, due to various factors, including the emergence of increasing numbers of immunocompromised people, the spread of pathogenic fungi is increasing worldwide, with simultaneous increases in fungal drug resistance and the incidence and mortality rates of fungal diseases (Rudramurthy et al., 2019; Suleyman and Alangaden, 2021; Chen et al., 2024; Denning, 2024; Ikuta et al., 2024). Not only have common opportunistic pathogenic fungi that cause invasive infections, such as *C. albicans*, *A. fumigatus*, and *Cryptococcus neoformans*, increased in recent years, the incidence of invasive infections caused by emerging pathogenic fungi, such as *C. glabrata*, *C. parapsilosis*, and *C. auris*, has also been increasing yearly (Colombo et al., 2017; Staniszewska, 2020). The slow progress in the development of vaccines and new antifungal drugs has intensified the difficulty of their clinical management, and fungal infections have become an increasingly important medical problem (Pathakumari et al., 2020; Zhang et al., 2021). Therefore, the development of new broad-spectrum antifungal drugs have become urgent. In a previous study, we confirmed that SNH exerted therapeutic antifungal activity against systemic *A. fumigatus* infection *in vivo* (Zhang et al., 2022). In the present study, the antifungal activity of SNH against *A. flavus* was investigated both *in vitro* and *in vivo*.

*Aspergillus* spores are internalized and invade the lung epithelial cells, forming hyphae in the lung tissue. These produce various toxins and proteases, trigger the host’s immune responses and ultimately lead to IPA (Lamoth and Calandra, 2022). In addition to *A. fumigatus*, *A. flavus* also contributes to IPA, particularly in Asian countries (Yang et al., 2021; Heylen et al., 2024). In recent years, the incidence of IPA has been increasing. Moreover, up to 20% of *Aspergillus* isolates worldwide have shown resistance to commonly used clinical antifungal drugs (Rudramurthy et al., 2019; Guegan et al., 2021). Fortunately, the 12 clinical *A. flavus* strains tested here showed no drug resistance to ITR, VRC, or AmB (Table 1). Moreover, they were also sensitive to SNH. However, the SNH results are still significantly higher than those of standard antifungals drugs for *Aspergillus*. Our previous analysis of the antifungal mechanism of SNH showed that SNH targets the cell membrane by inhibiting ergosterol synthesis in *A. fumigatus* (Zhang et al., 2022). However, further investigations are required to clarify the underlying mechanism of SNH against *A. flavus*.

IPA is one of the most serious fungal diseases of humans and can be life-threatening. The inhalation of the asexual spores of *Aspergillus* species can produce localized granulomas and may cause widespread suppurative pneumonia accompanied by abscess formation and acute coagulative necrosis (Cadena and Patterson, 2021). Infection may also be accompanied by necrotizing vasculitis, thrombosis, and bacterial thrombi (Chen et al., 2024). Toll-like receptors are the essential receptors involved in the immune response to *Aspergillus* infections *in vivo* (Balloy et al., 2005). They participate in the regulation of the expression of inflammatory genes, including *IL-6*, *IL-1β*, and *TNF-*α (Xie et al., 2022). These cytokines play a crucial role in protecting host from infection, especially in the earlier communication to coordinate the various aspects of immune system (Shankar et al., 2024). As in previous studies, inflammatory genes including *IL-1β* and *TNF-*α were upregulated in the IPA model mice (Mehrad et al., 1999). Moreover, the immunological response was stronger in the immunocompetent mice than that in the healthy mice (Anand et al., 2013). Consistent with our study of the anti-*A. fumigatus* infection (Zhang et al., 2022) and itraconazole application, treatment with antifungal agent SNH significantly reduced the fungal load of *A. flavus* (Figure 3). In addition, the treatment with SNH was administered by gastric gavage. Therefore, the SNH or the metabolic product of SNH against the *A. flavus* lung infection may be through Gut-lung axis. Moreover, similar to other plant secondary metabolites (Zhang et al., 2016; Zhu et al., 2019; Wang et al., 2019; Zhou et al., 2023) and itraconazole (Choi et al., 2010), SNH can also be used as an immunomodulatory agent and exerts anti-inflammatory effects (Figures 4, 5). Interestingly, after treatment with SNH in invasive aspergillosis mice, the levels of IFN-γ and TNF-α were unchanged with respect to those in the sham control (Zhang et al., 2022). Our unpublished investigation data also confirmed that SNH directly acts on the Toll-like receptors and effectively modulate immune responses through a threshold dose for anti-inflammatory effects. However, further investigations are required to clarify these mechanisms by which SNH acts against *A. flavus* from the host’s perspective.

## 5 Conclusion

The aim of the present study was to confirm the antifungal activity of SNH against *A. flavus*, both *in vitro* and *in vivo*. Although further investigations are required to confirm the antifungal mechanism involved, our results demonstrated that SNH exerted an anti-*A. flavus* effect. The data from this analysis of a mouse model of IPA supported that proposition that SNH presents a consistent with itraconazole application, exerts anti-inflammatory effects, and has potential utility in the treatment of *A. flavus* infections.

## Data Availability

The original contributions presented in the study are included in the article/Supplementary Material, further inquiries can be directed to the corresponding authors.
